# Tinnitus and Its Comorbidities: A Comprehensive Analysis of Their Relationships

**DOI:** 10.3390/jcm14041285

**Published:** 2025-02-15

**Authors:** Stefani Maihoub, Panayiota Mavrogeni, Viktória Molnár, András Molnár

**Affiliations:** 1 Maihoub ENT Clinic; Aliakmona Street 16, Cy-3117 Limassol, Cyprus; stefaniem-9@hotmail.com; 2 Tóth Ilona Health Service Clinical Medical Institute; Görgey Artúr tér 8, H-1212 Budapest, Hungary; panayiota.mavrogeni@icloud.com; 3 Department of Otorhinolaryngology and Head and Neck Surgery, Semmelweis University; Szigony u. 36, H-1083 Budapest, Hungary; molnar.viktoria@semmelweis.hu; 4 Protone Audio Kft., Opera Clinic; Lázár u. 4, H-1065 Budapest, Hungary

**Keywords:** tinnitus, comorbidities, Tinnitus Handicap Inventory, auditory impairment, hearing loss

## Abstract

**Objectives:** This study aimed to explore the demographic and clinical features of tinnitus individuals and analyse its correlation with associated comorbidities. **Methods:** The study population comprised 147 participants (66 men, 81 women; median age: 52 years) who experienced persistent tinnitus. Comprehensive assessments were carried out, including audiological examinations, scoring using the Tinnitus Handicap Inventory, and thorough medical evaluations. Statistical analyses were applied to explore the correspondences between tinnitus, hearing loss, and various comorbidities, including cardiovascular conditions, metabolic disorders, gastroesophageal reflux disease, autoimmune diseases, pulmonary diseases, and allergic rhinitis. **Results:** The analysis indicated a slight predominance of females, comprising 55.1% of the participants, with a median onset of tinnitus around the age of 50. Chronic tinnitus was noted, lasting approximately 46 months. Hearing loss was noted in 52.4% of patients, with bilateral tinnitus being the most prevalent type, affecting 44.2% of individuals. Dyslipidaemia was found to significantly predict bilateral tinnitus (*p* = 0.003*) and left-sided tinnitus (*p* = 0.023*). Additionally, atherosclerosis was associated with hearing impairment (*p* = 0.006*) and right-sided tinnitus (*p* = 0.044*). Dyslipidaemia was also significantly correlated with elevated intensity values (*p* = 0.04*). Furthermore, atherosclerosis was significantly associated with higher levels of hearing loss (*p* < 0.00001*). **Conclusions:** The study emphasises the complex nature of tinnitus and its links to cardiovascular, metabolic, and other comorbidities, highlighting the necessity for comprehensive, interdisciplinary management.

## 1. Introduction

Tinnitus is a prevalent and often debilitating condition marked by the sense of phantom sounds in the ears or head. Extensive research has focused on this issue in recent years. These phantom sounds can manifest as a ringing, buzzing, hissing, clicking, whistling, or whooshing sound, varying in intensity and pitch [1]. Primary tinnitus cases are often related to sensorineural hearing loss. These cases also encompass genetic hearing loss; for example, tinnitus may be associated with mutations in the WFS1 gene [2]. Recent research indicates that tinnitus is not just an ear-related issue; it is a complex condition linked to the central nervous system and often associated with multiple comorbidities. The prevalence of tinnitus worldwide varies significantly, ranging from 4% to 37%, depending on the population and criteria used in studies. In Europe, estimates fall between 9% and 29% [3]. For those affected, tinnitus can significantly reduce quality of life, with severe cases linked to depression, anxiety, and even suicidal thoughts [4]. Understanding the intricate relationship between tinnitus and its associated conditions is crucial for improving patient diagnostics and outcomes. These comorbidities can include cardiovascular problems, metabolic disorders, gastrointestinal diseases, autoimmune diseases, pulmonary diseases, and allergic rhinitis [3,5,6].

Studies on cardiovascular conditions related to tinnitus have identified atherosclerosis, hypertension, and dyslipidaemia as key factors. Atherosclerosis is a chronic inflammatory disease characterised by plaque build-up in the arteries and has been found to significantly contribute to tinnitus [5]. Research shows that individuals with tinnitus often have a higher degree of carotid artery atherosclerosis compared to those without the condition [7]. This association may be due to impaired blood flow and a reduced oxygen supply to the inner ear, which are essential for maintaining proper auditory function. When these systems are compromised, the likelihood of developing tinnitus increases. Hypertension is a major cardiovascular risk factor that has been closely associated with tinnitus [8]. High blood pressure can yield structural injuries to the sensitive components in the inner ear, potentially resulting in tinnitus. Additionally, treating hypertension with certain medications, like diuretics and calcium channel blockers, has been linked to a higher risk of developing tinnitus. Thus, a potential ototoxic effect of these medications should be considered, highlighting the importance of carefully selecting antihypertensive therapies for patients who are prone to tinnitus [9,10]. Moreover, dyslipidaemia, characterised by elevated levels of cholesterol and triglycerides in the bloodstream, is significant in developing tinnitus, according to the existing literature [11]. Inadequate oxygen delivery can hinder the function of antioxidant enzymes, resulting in the build-up of free radicals that can lead to oxidative injuries in the cochlea’s hair cells and the stria vascularis [12]. Additionally, hyperlipidaemia may compromise blood flow to the cochlea, increasing the risk of tinnitus. Notably, in some patients, tinnitus may be the first symptom of underlying atherosclerosis, highlighting the interconnected nature of these conditions.

Thyroid dysfunction, encompassing both hypothyroidism [13] and hyperthyroidism [14], has been linked to the development of tinnitus. While the exact mechanisms are not fully understood, changes in thyroid hormone levels may directly or indirectly impact the auditory system. Wang et al. suggested that imbalances in the sympathetic nervous system, caused by disruptions in thyroid hormones, could lead to damage in the cochlea, resulting in tinnitus. This damage may be worsened in individuals with hypothyroidism, as insufficient oxygen supply to the cochlea further contributes to auditory dysfunction [13]. The common mechanisms behind these conditions—such as impaired blood flow, reduced oxygen delivery, and oxidative stress—highlight a shared pathway in tinnitus development.

Kang et al. [15] uncovered an intriguing link between tinnitus and gastroesophageal reflux disorder (GERD). Their study indicates that acidic components may disturb the balance of the middle and inner ear by impacting the function of the Eustachian tube. Additionally, one hypothesis that has yet to be explored is that the reflux of acidic gastric contents into the laryngopharynx could lead to irritation and inflammation, activating the vagus nerve and resulting in the perception of tinnitus. Furthermore, metabolic conditions, such as diabetes mellitus (DM), have also been strongly associated with tinnitus. Hyperglycaemia can cause microvascular damage and neuropathy, which may impair the function of the auditory system [16]. Studies state that approximately 26.4% of DM patients experience tinnitus, highlighting the significant overlap between these conditions [17].

Allergic rhinitis is a common inflammatory condition affecting the nasal mucosa and may also be associated with tinnitus. The release of inflammatory mediators, such as histamine, can increase blood flow and vascular permeability in the inner ear [18], potentially contributing to the perception of tinnitus. Furthermore, autoimmune diseases, e.g., rheumatoid arthritis, Sjögren’s syndrome, and systemic lupus erythematosus are linked to a higher risk of developing tinnitus [19]. Additionally, pulmonary conditions, including chronic obstructive pulmonary disease (COPD) and asthma [20], along with interstitial lung disease, further illustrate the systemic nature of tinnitus. Psychiatric comorbidities, such as depression and anxiety, are frequently reported in individuals with tinnitus. This may be attributed to glucocorticoid-mediated glutamate release in the cochlea, leading to central nervous system responses affecting tinnitus. Understanding these correlations is crucial when considering tinnitus treatment options [21]. The complex nature of tinnitus, along with its related conditions, requires a thorough and multidisciplinary approach to management.

Given the high prevalence of tinnitus alongside various systemic comorbidities, this article explores the connections between tinnitus and other health conditions. It examines its associations with diseases such as atherosclerosis, hypertension, thyroid dysfunction, and DM, among others. By clarifying these links, we aim to emphasise the importance of a holistic approach to managing tinnitus and enhancing patient outcomes.

## 2. Materials and Methods

### 2.1. Study Population

This retrospective study involved 147 patients (66 men, 81 women; median age: 52 years) who experienced persistent subjective tinnitus, encompassing both acute and chronic cases. These patients were examined between October 2022 and November 2024. All participants received an otorhinolaryngological examination, including tympanometry and acoustic reflex testing, conducted by the same specialist (A.M.) at the Opera Clinic Hearing and Balance Centre in Budapest, Hungary. They also received audiological assessments and tinnitometry, along with comprehensive laboratory tests, hormonal panels, and medical imaging (such as brain MRI and carotid Doppler ultrasonography). Diagnosis of associated medical conditions was made by qualified specialists and was taken from the participants’ detailed case history. When considering comorbidities, the following conditions were taken into account: hypertension, DM (both type 1 and type 2), thyroid dysfunctions (including both hyperthyroidism and hypothyroidism) GERD, dyslipidaemia, atherosclerosis, allergic rhinitis confirmed by allergy testing (Prick test and total IgE levels over 100 IU/mL), pulmonary diseases (such as COPD and asthma), psychiatric disorders (including anxiety and depression), and autoimmune diseases (e.g., systemic lupus erythematosus, rheumatoid arthritis, and Sjögren’s syndrome). Systemic lupus erythematosus [22], rheumatoid arthritis [23], and Sjögren’s syndrome [24] were diagnosed based on comprehensive immunological assessments. Anxiety and depression were evaluated using the criteria specified in the *Diagnostic and Statistical Manual of Mental Disorders*, *Fifth Edition* (DSM-5) during a psychiatric assessment [25]. DM was diagnosed by a diabetologist in each case, and both type 1 and type 2 DM were considered. The diagnostic criteria for DM include a haemoglobin A1C (HbA1c) cut-off value of 48 mmol/L (6.5%). Additionally, a fasting glucose level of 7.00 mmol/L or higher, or a random venous glucose level of 11.1 mmol/L or higher, is also used for diagnosis, in accordance with WHO recommendations [26]. Thyroid dysfunctions were evaluated through a thorough endocrinological examination, which included assessments for both hyperthyroidism and hypothyroidism, such as Hashimoto’s thyroiditis and Graves–Basedow disease. The primary criteria for diagnosing Hashimoto’s thyroiditis include the presence of hypothyroidism, an elevated TSH level (greater than 4.5 mIU/L), which may occur alongside low levels of thyroid hormones (T3 and T4), and the detection of circulating antibodies against thyroid antigens, specifically thyroperoxidase and thyroglobulin. Additionally, a thyroid ultrasound may reveal an enlarged thyroid or the presence of goitre [27]. These examinations also aided in detecting hyperthyroidism (suppressed TSH and elevated T3 and T4 levels), including Graves–Basedow disease. The T3/T4 ratio can be utilised for the differential diagnosis of Graves–Basedow disease, where a FT3/FT4 ratio greater than 0.3 suggests Graves’ disease and helps differentiate it from thyroiditis-induced thyrotoxicosis. To diagnose Graves’ disease, the TSH-receptor antibody (TRAb) test also exhibits both high sensitivity and specificity [28]. Hypertension was diagnosed through comprehensive cardiological or internal medical examinations. Hypertension is defined as a systolic blood pressure of 140 mmHg or higher and/or a diastolic blood pressure higher than 90 mmHg, as measured by medical staff and confirmed through 24 h blood pressure monitoring [29]. Atherosclerosis was assessed using carotid and vertebral Doppler ultrasonography, based on the report from a radiological specialist. For GERD, indicator signs were identified through laryngoscopy, and a gastroenterological examination confirmed the final diagnosis. The Hungarian Tinnitus Handicap Inventory (THI) questionnaire was used to assess the influence of tinnitus on daily functioning. All patients provided their consent in writing. Exclusion criteria included incomplete medical data, patients under 18 years of age, and a lack of participation in the investigation. The investigation adhered to the Declaration of Helsinki and received approval from the Hungarian ETT TUKEB (approval number: BM/29864–1/2024, approval date: 9 December 2024).

### 2.2. Tinnitus Handicap Inventory

The previously validated Hungarian THI questionnaire was used to assess participants’ self-reported tinnitus severity. The THI consists of 25 items organised into three categories as follows. The emotional scale consists of nine items that focus on various issues, such as depression, anxiety, or frustration. The functional scale relates to everyday activities, including interpersonal relationships, household management, and stress counselling. The catastrophic category focuses on experiences related to feelings of severe illness and loss of control. The total score is calculated by adding together the points from all three subscales. According to the scores, tinnitus severity is classified into five classes, i.e., ‘no handicap’ (0–16 points), ‘mild’ (18–36), ‘moderate’ (38–56), ‘severe’ (58–76), and ‘catastrophic’ (78–100) tinnitus handicaps [30,31].

### 2.3. Audiological Examination and Tinnitus Pitch-Matching

Pure-tone audiometric testing was performed by applying a GSI 61 Audiometer (Grason Stadler, Inc., Milford, CT, USA) in an acoustic isolation chamber. Prior to audiometric testing, an otorhinolaryngologist performed physical examinations and tympanometry on the external and middle ears of each participant. The assessment involved testing pure-tone air conduction (from 125 to 8000 Hz) and bone conduction (from 250 to 4000 Hz) using headphones and a vibrator applied to the mastoids, respectively. Bone conduction testing was conducted using masking when necessary. Following the recommendations of the Committee on Hearing and Equilibrium from the American Academy of Otolaryngology—Head and Neck Surgery, averages from pure-tone audiometry were established, and sensorineural hearing loss was clearly defined [32].

All participants were also evaluated to assess the frequency and loudness of their tinnitus sounds. The pitch-match frequency was determined using a range of frequencies from 125 Hz to 8000 Hz. The evaluations were performed for the right, left, or both sides, based on where the symptoms were located.

### 2.4. Statistical Analysis

Statistical analyses were conducted using the Statistical Package for the Social Sciences (SPSS) version 25.0 (IBM Corporation, Armonk, NY, USA). To assess whether the sample data were drawn from a normally distributed population, the Shapiro–Wilk test was conducted, which revealed a non-normal distribution of the data. Continuous variables were reported as medians, accompanied by their interquartile ranges (IQRs). The Mann–Whitney *U* test was utilised to determine if there were statistically significant differences between the groups. Additionally, a logistic regression algorithm was used to analyse the effects of various parameters on tinnitus. Furthermore, Spearman’s correlation test was used to assess the relationships among the variables. The significance level was set at *p* < 0.05.

## 3. Results

The study population’s characteristics were analysed, and the outcomes are summarised in Table 1.

The results presented in Table 1 show a slight female predominance in the current tinnitus population, with females making up 55.1% of the total. Additionally, the median age indicates that the occurrence of tinnitus tends to increase around the age of 50. Increasing age is also crucial when considering comorbidities; as individuals age, the likelihood of developing comorbidities increases significantly. The median onset of tinnitus symptoms was approximately 16 months, indicating the emergence of chronic tinnitus in many cases. Hearing loss occurred concomitantly in about half of the current population. This refers to a substantial percentage of tinnitus associated with sensorineural hearing loss. Bilateral tinnitus was the most common type of tinnitus observed based on symptom location, followed by left-sided tinnitus. The phenomenon of left-sided tinnitus may be attributed to the higher prevalence of right-handed individuals in the population. Most cases showed mild tinnitus handicap according to median total THI scores. Hearing levels mostly indicated mild hearing loss, with tinnitus occurring more frequently at middle and higher frequencies. The presence of tinnitus at these frequencies highlights the link between tinnitus and hearing loss. Upon analysing the relationships between the aforementioned parameters, a significant correlation was discovered between the onset of tinnitus and the participants’ ages (rho = 0.171, *p* = 0.038*, according to Spearman’s correlation test). This result suggests that longer-lasting tinnitus symptoms are associated with increasing age. Additionally, there was a significant relationship between age and hearing levels (rho = 0.451, *p* = 0.000*), indicating that the ageing process significantly affects hearing. Furthermore, a notable relationship was observed between tinnitus onset and hearing levels (rho = 0.198, *p* = 0.026*), indicating that poorer hearing levels are associated with longer durations of tinnitus.

**Figure 1:**
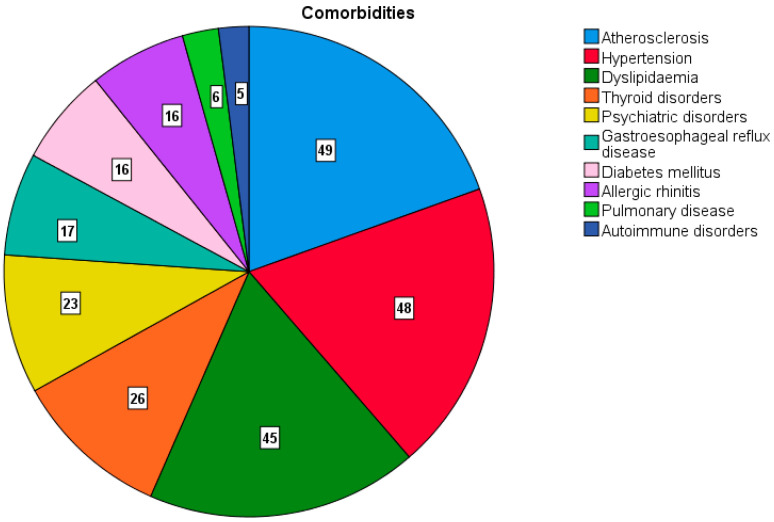
The distribution of certain comorbidities within the study population. The numbers in the pie chart represent the number of patients.

As illustrated in Figure 1, the most prevalent co-occurring comorbidities in this population were atherosclerosis (*n* = 49, 33.3%), hypertension (*n* = 48, 32.7%), and dyslipidaemia (*n* = 45, 30.6%). The increased prevalence of these co-occurring comorbidities emphasises the strong link between tinnitus and vascular risk factors. The next most prevalent group of comorbidities included thyroid disorders (*n* = 26, 17.7%), psychiatric disorders (*n* = 23, 15.6%), and GERD (*n* = 17, 11.6%). While DM (*n* = 16, 10.9%), allergic rhinitis (*n* = 16, 10.9%), pulmonary diseases (*n* = 6, 4.1%) and autoimmune disorders (*n* = 5, 3.4%) showed lower rates of co-occurrence.

The data summarised in Table 2 show that dyslipidaemia is a significant predictor of both bilateral tinnitus (*p* = 0.003) and left-sided tinnitus (*p* = 0.023). This finding is particularly important for bilateral tinnitus, as it highlights a markedly increased risk of developing this condition in individuals with dyslipidaemia, emphasising the impact of vascular risk factors on tinnitus development. However, atherosclerosis significantly predicted sensorineural hearing loss (*p* = 0.006) and right-sided tinnitus (*p* = 0.044). This finding emphasises the significance of atherosclerosis in developing sensorineural hearing loss linked to tinnitus, likely due to disruptions in the blood supply to the inner ear. Right-sided tinnitus was also associated with thyroid dysfunction (*p* = 0.009), indicating potential autoimmune mechanisms involved in the development of unilateral tinnitus. Psychiatric comorbidities significantly impacted the development of moderate to severe tinnitus (*p* = 0.001), suggesting that individuals with these conditions face a higher risk of experiencing severe tinnitus-related difficulties.

**Figure 2:**
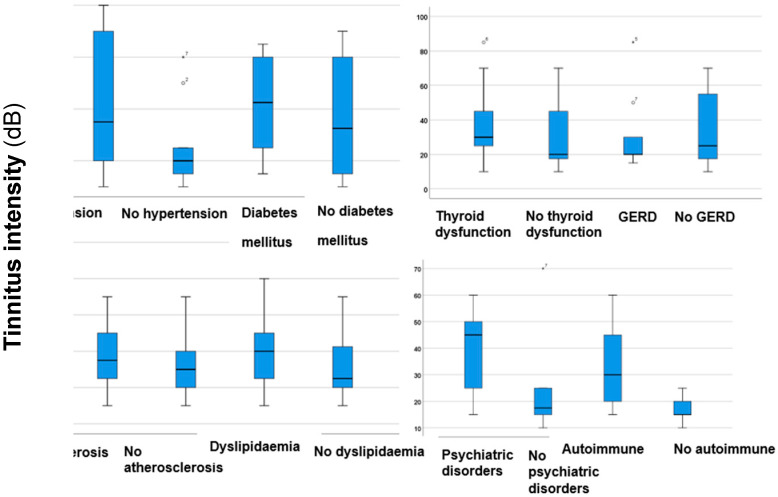
Boxplots illustrating tinnitus intensities in participants with hypertension, DM, thyroid disorders, atherosclerosis, dyslipidaemia, psychiatric disorders, GERD, and autoimmune diseases, as well as those without these comorbidities. The boxes indicate the interquartile range of the data, while the whiskers show the lower and upper quartiles. The black line that separates the boxes marks the median values. Differences between groups were analysed utilising the Mann–Whitney *U* test. DM = diabetes mellitus; GERD = gastroesophageal reflux disease. The significant results (*p* < 0.05) are indicated with an asterisk (*). The degrees with the superscript numbers indicate the outliers.

**Figure 3:**
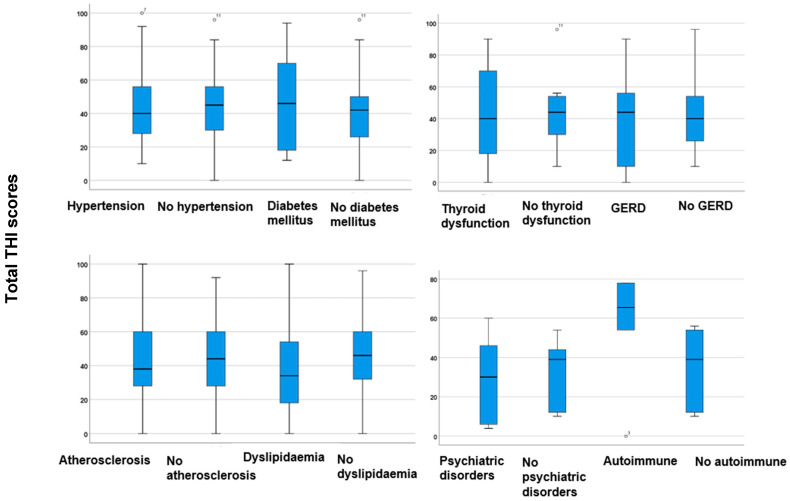
Boxplots illustrating total THI scores in participants with hypertension, DM, thyroid disorders, atherosclerosis, dyslipidaemia, psychiatric disorders, GERD, and autoimmune diseases, as well as those without these comorbidities. The boxes indicate the interquartile range of the data, while the whiskers show the lower and upper quartiles. The black line that separates the boxes marks the median values. Differences between groups were analysed utilising the Mann–Whitney *U* test. DM = diabetes mellitus; GERD = gastroesophageal reflux disease; THI = Tinnitus Handicap Inventory The degrees with the superscript numbers indicate the outliers.

As a further step in the investigation, the differences in total THI scores, tinnitus intensities, and hearing levels were analysed, and the results are given in Figure 2 and Figure 3.

As depicted in Figure 2, no statistically significant difference was observed in tinnitus intensities between participants with hypertension and those without (*p* = 0.26, *z*-score: 1.12; Mann–Whitney *U* test). However, patients with hypertension tended to have higher tinnitus intensity values. This result indicates that hypertension may have an impact on the volume of tinnitus sounds, making them louder. There were no significant differences or trends between the groups concerning thyroid dysfunction (*p* = 0.85, *z*-score: 0.17), GERD (*p* = 0.28, *z*-score: 1.08), and atherosclerosis (*p* = 0.267, *z*-score: −1.11). This indicates that acidic components, changes in thyroid hormones, and artery narrowing or blockage do not significantly affect the loudness of tinnitus. DM, psychiatric disorders, and autoimmune diseases showed no significant differences in tinnitus intensities (*p* > 0.05). However, patients with dyslipidaemia exhibited significantly higher (*p* = 0.04*, *z*-score: −1.9) tinnitus intensities than those without dyslipidaemia. These findings emphasise the considerable influence considering dyslipidaemia on the onset of louder tinnitus, which can be more distressing for those affected. Additionally, the potential effects of hypertension should also be considered.

Figure 3 illustrates the total THI scores, considering various comorbidities.

In analysing the total THI scores, no statistically significant differences were observed between participants with comorbidities and those without, as determined by the Mann–Whitney *U* test (*p* > 0.05). This indicates that none of the co-occurring comorbidities notably affect the tinnitus severities reported by the THI. In other words, these comorbidities do not lead to a significant increase in tinnitus severity. However, there was a tendency for higher total THI scores among participants with DM, thyroid dysfunctions, and autoimmune diseases (Figure 3). The results indicate that high blood glucose levels, thyroid dysfunction, and autoimmune disorders may affect the severity of tinnitus, warranting further investigation.

**Figure 4:**
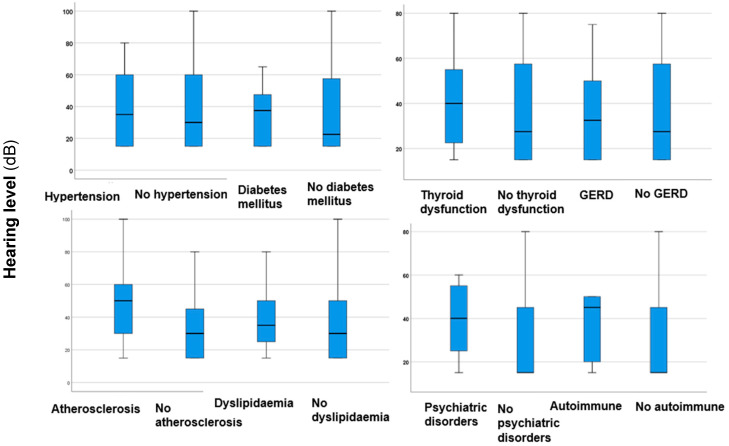
Boxplots illustrating hearing levels in participants with hypertension, DM, thyroid disorders, atherosclerosis, dyslipidaemia, psychiatric disorders, GERD, and autoimmune diseases, as well as those without these comorbidities. The boxes indicate the interquartile range of the data, while the whiskers show the lower and upper quartiles. The black line that separates the boxes marks the median values. Differences between groups were analysed utilising the Mann–Whitney *U* test. The significant results (*p* < 0.05) are indicated with an asterisk (*). GERD = gastroesophageal reflux disease.

In analysing the differences in hearing levels among comorbidities, a statistically significant difference (*p* < 0.00001*, *z*-score: −4.209, Mann–Whitney *U* test) was found regarding atherosclerosis, showing higher values in patients with this condition. This means that atherosclerosis, which causes artery narrowing or blockage, can lead to more severe sensorineural hearing loss, possibly due to disrupted blood flow in the inner ear. No statistically significant differences (*p* > 0.05) or trends were observed regarding other comorbidities (Figure 4). This indicates that these comorbidities do not increase the risk of more severe hearing loss.

## 4. Discussion

The analysis of the study population offers valuable knowledge of the demographic and clinical features of individuals with tinnitus and their associated comorbidities. The study revealed a slight female predominance, with 55.1% of the participants being female. This finding suggests that women may be marginally more affected by tinnitus than men, which is consistent with other studies indicating sex-related differences in the perception of tinnitus [33]. Additionally, participants’ median age indicates that the occurrence of tinnitus tends to increase around the age of 50, highlighting the impact of age-related auditory changes. This reinforces the notion that the occurrence of tinnitus tends to rise as people get older [34]. Notably, on average, individuals experienced tinnitus symptoms for a median duration of about 16 months, suggesting that a significant portion of the population endures chronic tinnitus, which necessitates long-term management strategies.

Sensorineural hearing loss was observed in approximately half of the participants, indicating a strong association between tinnitus and changes in hearing. This underscores the importance of comprehensive audiological evaluations for these patients. Tan et al. [35] studied the patterns of hearing loss associated with tinnitus, noting that individuals with tinnitus generally demonstrate better hearing levels at low frequencies but worse levels at high frequencies compared to the control group. The hearing assessments revealed that ‘mild’ hearing loss was the most frequently observed auditory profile, with tinnitus frequently occurring at middle and higher frequencies. This pattern aligns with typical age-related hearing loss, further strengthening the connection between tinnitus and hearing deterioration. Other authors have examined age-related differences in hearing thresholds. Research indicates that younger tinnitus patients generally have lower hearing thresholds compared to those without tinnitus. In contrast, older patients show a wider range of results [36,37]. Authors have discussed and emphasised the influence of degenerative bony conditions on the development or worsening of tinnitus. Vîrlan et al. [38] reviewed how biomechanical changes in the middle and inner ear are related to degenerative bony alterations in the temporal component of the temporomandibular joint. In terms of symptom laterality, bilateral tinnitus was the most common type reported, followed by tinnitus affecting the left ear. This prevalence of bilateral symptoms may suggest the presence of systemic or generalised auditory pathologies rather than localised conditions.

The THI scores in the current study are promising, indicating that for most individuals, tinnitus does not significantly impact daily functioning. However, this conclusion can be debated, as other researchers have demonstrated that tinnitus can profoundly affect quality of life in adults [39]. Our findings also suggest that even mild tinnitus can influence quality of life, highlighting the need for tailored interventions to reduce distress. However, it is important to note that the intensity of self-perceived tinnitus varies among individuals and depends on multiple factors. Additionally, even tinnitus cases that have a mild impact on daily functioning should be considered and carefully assessed.

This study found significant correlations between several key parameters. The onset of tinnitus was positively correlated with age, suggesting that longer durations of tinnitus are associated with increasing age. Additionally, a strong correlation was identified between age and hearing loss, supporting the earlier mentioned expectation. Furthermore, the onset of tinnitus was significantly linked to hearing levels, indicating that hearing impairment may play a role in the occurrence of tinnitus. A prior investigation also found that the prevalence of tinnitus increases with age [40]. Age-related hearing loss has been thoroughly examined in the existing literature, highlighting a multifactorial background. This includes factors such as noise exposure, ototoxic agents, genetic predispositions, metabolic diseases, and lifestyle choices [41]. Sensorineural hearing loss, one of the most critical factors for developing tinnitus, has also been implicated [42].

Our study provided further analysis of comorbidities related to tinnitus. The most common cooccurring conditions we found were atherosclerosis (33.3%), hypertension (32.7%), and dyslipidaemia (30.6%). Lee et al. [43] demonstrated that dyslipidaemia, characterised by hypertriglyceridemia and a high total cholesterol/HDL-cholesterol ratio, was significantly related to an increased OR of tinnitus in older individuals. Following these, we observed thyroid disorders (17.7%), psychiatric disorders (15.6%) and GERD (11.6%). Lower rates of cooccurrence were noted for DM (10.9%), allergic rhinitis (10.9%), pulmonary diseases (4.1%), and autoimmune disorders (3.4%). Our findings indicated that dyslipidaemia is a significant predictor of both bilateral and left-sided tinnitus, suggesting that individuals with dyslipidaemia are at a higher risk of experiencing tinnitus, particularly bilaterally. One potential explanation for dyslipidaemia in tinnitus development is the obstruction of capillaries in rigid vessels within the inner ear. This can lead to biochemical changes in the endolymph and result in inner ear ischaemia [44]. The decreased blood flow to the stria vascularis can cause sensorineural hearing loss and lead to tinnitus [45]. The high prevalence of sensorineural hearing loss associated with tinnitus supports this observation. Moreover, high levels of blood lipids lead to lipid deposition in the cell membranes of outer hair cells, causing chronic hypoxia and disrupted metabolism [46].

In contrast, atherosclerosis was significantly linked to sensorineural hearing loss and right-sided tinnitus. Additionally, the link between thyroid disorders and right-sided tinnitus highlights the importance of metabolic conditions in influencing tinnitus symptoms. The exact mechanism underlying the relationship between tinnitus and thyroid dysfunction is still not fully understood. One possible explanation is the influence of thyroid hormones on the sympathoadrenal system and the resulting impairment of cochlear blood flow [13]. Moreover, it has been proposed that thyroid hormones are involved in the maturation and development of the central nervous system [47] as well as the organ of Corti [48]. Moreover, psychiatric comorbidities were found to have a substantial impact, contributing to moderate to severe tinnitus, which highlights the importance of addressing mental health in tinnitus management.

Investigating the loudness of tinnitus revealed that hypertension was not significantly associated with loudness differences. However, there was a tendency for higher tinnitus intensity values in patients with hypertension. Hypertension can damage the inner ear through two main mechanisms: impaired cochlear microcirculation and the ototoxic effects of certain antihypertensive drugs, such as furosemide and beta blockers [49]. A previous investigation observed that tinnitus has been linked to hypertension in younger individuals, specifically those aged 20 to 29 [50]. A different study found that adults with hypertension have higher rates of hearing loss [51]. A longitudinal study found that patients with hypertension had poorer hearing. However, after adjusting for factors such as age, sex, DM diagnosis, and noise exposure, the differences in hearing became non-significant. Additionally, higher rates of tinnitus were observed among these patients, but this difference also became insignificant once age was taken into account [52]. Additionally, a study found that patients with tinnitus exhibited higher rates of masked hypertension. Therefore, tinnitus may serve as an early indicator of hypertension [53]. Dyslipidaemia was the only comorbidity significantly linked to higher tinnitus intensities, suggesting that changes in lipid metabolism may contribute to the perception of louder tinnitus. To our knowledge, no research has been published on the correlation between tinnitus intensity and comorbidities. Analysing the impact of tinnitus on daily functioning showed no significant differences in total THI scores between participants with and without comorbidities. However, individuals with DM, thyroid dysfunction, and autoimmune diseases tended to have higher THI scores, indicating a possible additive effect of these conditions on the severity of tinnitus. These correlations may be potential topics for future research plans. The link between tinnitus, hearing loss, and DM may be attributed to the neuropathy and microangiopathy caused by hyperglycaemia. Hyperglycaemia adversely affects neural cells through various mechanisms, including the thickening of capillary basement membranes, endothelial cell hyperplasia, and a reduction in Na + K + ATPase activity [17].

Finally, the analysis of hearing-level differences across various comorbidities revealed a significant association between atherosclerosis and increased levels of hearing loss. This finding underscores the vascular factors contributing to auditory dysfunction and highlights the importance of cardiovascular health for reducing the risk of hearing loss and tinnitus. Additionally, Wattawar et al. [54] examined the link between coronary artery disease and hearing loss, discovering a strong correlation with audiometric results and noting hearing loss across all frequency ranges.

To summarise the findings of this investigation, it is crucial to take comorbidities into account when managing tinnitus. The results highlight the need for a comprehensive, multidisciplinary approach to tinnitus management. This approach should include not only assessments related to ear, nose, and throat care but also the diagnosis and treatment of comorbid conditions to help alleviate tinnitus symptoms. Additionally, further research into the treatment of comorbidities in individuals with tinnitus is warranted.

While this study provides valuable insights, it is important to acknowledge several limitations. The study’s cross-sectional design limits the ability to demonstrate a causal relationship between tinnitus and its associated comorbidities. Additionally, only the presence of comorbidities was considered, while specific blood pressure or laboratory testing values were not analysed. Additional investigation is necessary to explore these relationships and confirm any potential causes. Additionally, the study population may not accurately represent the general population, as participants were drawn from specific clinical settings, which could lead to selection bias. Moreover, the significant variation in the duration of tinnitus, whether acute or chronic, may introduce bias into the results. The effects of treatments for comorbidities could not be analysed either. Lastly, while significant correlations are identified, the effect sizes for some associations are relatively small, suggesting that other unmeasured factors may also contribute to the observed relationships. Improving future research can lead to a deeper understanding of tinnitus and its complexities.

## 5. Conclusions

These findings collectively highlight the complex nature of tinnitus, emphasising the relationships between age, hearing loss, comorbidities, and symptom severity. The results underscore the necessity for comprehensive, age-sensitive approaches in managing tinnitus, as well as the need for further research into interventions that address these issues.

## Figures and Tables

**Table 1:** Participants general characteristics. IQR = interquartile range; Q1 = first quartile; Q3 = third quartile; SD = standard deviation; THI = Tinnitus Handicap Inventory. Hearing loss, specifically when it exceeds 20 dBs, is defined as a clinically significant loss of hearing.

Parameter	
Age (median years, IQR; Q1–Q3)	52 (21; 43–64)
Sex (men/women)	66/81
Symptom onset (median months, IQR; Q1–Q3)	16 (55; 5–60)
Tinnitus location	
Right (*n*, %)	34 (23.1%)
Left (*n*, %)	48 (32.6%)
Bilateral (*n*, %)	65 (44.21%)
Hearing loss (*n*, %)	77 (52.38%)
Hearing level (median dB, IQR; Q1–Q3)	30 (30; 15–45)
Tinnitus intensity (median dB, IQR; Q1–Q3)	30 (30; 20–50)
Tinnitus pitch (median Hz, IQR; Q1–Q3)	4000 (6000; 2000–8000)
Total THI scores (median, IQR; Q1–Q3)	44 (38; 28–66)

**Table 2:** Logistic regression analysis in predicting different dependent variables. CI = confidence interval; OR = odds ratio; Std. = standard. The significant results (*p* < 0.05) are indicated with an asterisk (*). Only results that were statistically significant were included in the table.

Dependent	Predictor	*β*	Std. Error	*p*-Value	OR	95% CI (Lower Bound)	95% CI (Upper Bound)
Bilateral tinnitus	Dyslipidaemia	−1.282	0.428	0.003 *	0.278	0.120	0.642
Sensorineural hearing loss	Atherosclerosis	1.400	0.513	0.006 *	4.054	1.483	11.086
Moderate to severe tinnitus	Psychiatric comorbidities	2.184	0.687	0.001 *	8.881	2.312	34.123
Right-sided tinnitus	Thyroid dysfunction	−2.430	0.933	0.009 *	0.088	0.014	0.548
	Atherosclerosis	1.038	0.516	0.044 *	2.823	1.026	7.766
Left-sided tinnitus	Dyslipidaemia	0.927	0.406	0.023 *	2.527	1.140	5.605

## Data Availability

The data presented in this study are available on request from the corresponding author due to reasonable request.
